# Accuracy of Smartwatch Pulse Oximetry Measurements in Hospitalized Patients With Coronavirus Disease 2019

**DOI:** 10.1016/j.mcpdig.2024.02.001

**Published:** 2024-02-26

**Authors:** Kevin Rajakariar, Paul Buntine, Andrew Ghaly, Zheng Cheng Zhu, Vihangi Abeygunawardana, Sarah Visakhamoorthy, Patrick J. Owen, Shaun Tham, Liam Hackett, Louise Roberts, Jithin K. Sajeev, Nicholas Jones, Andrew W. Teh

**Affiliations:** a Department of Cardiology, Eastern Health, Melbourne, Victoria, Australia; b Department of Cardiology, Austin Health, Melbourne, Victoria, Australia; c Department of Emergency Medicine, Eastern Health, Melbourne, Victoria, Australia; d Eastern Health Clinical School, Monash University, Melbourne, Victoria, Australia

## Abstract

**Objective:**

To assess the ability of 2 commercially available smartwatches to accurately detect clinically significant hypoxia in patients hospitalized with coronavirus-19 (COVID-19).

**Patients and Methods:**

A prospective multicenter validation study was performed from November 1, 2021, to August 31, 2022, assessing the Apple Watch Series 7 and Withings ScanWatch inbuilt pulse oximetry, against simultaneous ward-based oximetry as the reference standard. Patients hospitalized with active COVID-19 infection not requiring intensive care admission were recruited.

**Results:**

A total of 750 smartwatch pulse oximetry measurements and 400 ward oximetry readings were successfully obtained from 200 patients (male 54%, age 66±18 years). For the detection of clinically significant hypoxia, the Apple Watch had a sensitivity and specificity of 34.8% and 97.5%, respectively with a positive predictive value of 78.1% and negative predictive value of 85.6%. The Withings ScanWatch had a sensitivity and specificity of 68.5% and 80.8%, respectively with a positive predictive value of 44.7% and negative predictive value of 91.9%. The overall accuracy was 84.9% for the Apple Watch and 78.5% for the Withings ScanWatch. The Spearman rank correlation coefficients reported a moderate correlation to ward-based photoplethysmography (Apple: r_s_=0.61; Withings: r_s_=0.51, both *P*<.01).

**Conclusion:**

Although smartwatches are able to provide SpO_2_ readings, their overall accuracy may not be sufficient to replace the standard photoplethysmography technology in detecting hypoxia in patients with COVID-19.

The uptake of smartwatch technology is becoming ubiquitous with more than 200 million individuals owning a smartwatch worldwide, and wearable technology is currently estimated to be a $43 billion market.^1^ Many consumers use smartwatch technology for its ability to monitor biometric data, and more recently the detection of arrhythmias. The recent addition of inbuilt pulse oximetry has become more relevant in the setting of the coronavirus disease 2019 (COVID-19) pandemic.^2^ Detection of blood oxygen (SpO_2_) levels using standard photoplethysmography (PPG) has been essential in the monitoring and management of patients infected with COVID-19 both at home and in hospital.^3^ Although PPG oximetry is the accepted standard for monitoring blood oxygen levels, smartwatch manufacturers and recent studies have promised consumers medical grade accuracy of blood oximetry readings.^4^ However, there is a paucity of data assessing the accuracy of smartwatch-derived pulse oximetry readings, especially in patients with COVID-19. We assessed the ability of 2 commercially available smartwatches to accurately detect clinically significant hypoxia in patients hospitalized with COVID-19, compared with the reference standard ward-based PPG readings.

## Methods

### Study Design

A prospective multi0center validation study was performed across 2 tertiary university hospitals (Austin Health, Victoria, Australia and Eastern Health, Victoria, Australia) between November 1, 2021, and August 31, 2022, where patients hospitalized with COVID-19 infection, not requiring intensive care, were invited to participate. Patients were excluded if they had severe COVID-19 infection requiring intensive care unit admission or were unable to consent. Informed consent was obtained with an electronic signature on a digital consent form to reduce transmission of infection between patients and medical staff. The baseline demographic characteristic data and clinical data, including Fitzpatrick skin color, hemoglobin, pre-existing peripheral arterial disease, or lung disease were recorded. The Fitzpatrick scale evaluates the color of skin and estimates its response to ultraviolet light using a numerical rating between 1 and 6, with 1 being lighter and more prone to sunburn and 6 being darker and less likely to develop sunburn.^5^ Lung disease was defined as any interstitial lung disease or chronic obstructive pulmonary disease confirmed on spirometry, or radiologically on computed tomography investigations. Peripheral arterial disease was defined as radiologically confirmed presence of atherosclerotic disease in the peripheral arteries. The institutional review boards for both institutions provided ethics approval for this study (LR21-045-80974; HREC/81563/Austin-2022).

**Figure 1:**
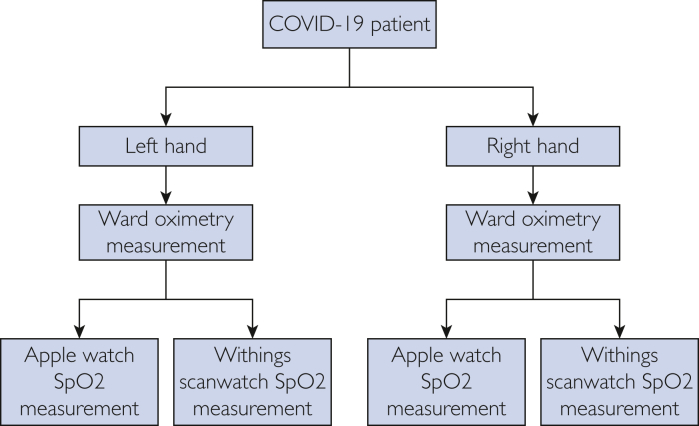
Protocol for obtaining smartwatch oximetry readings compared with ward-based PPG monitor.

A therapeutic goods administration approved hospital-grade pulse oximeter (Welch Allyn Connex Spot Monitor) was placed on the index finger of the left hand to obtain a reference standard oxygen saturation reading. Immediately after this, an Apple Watch (AW) Series 7 (Apple Inc) and then a Withings ScanWatch (WS) (Withings) were consecutively placed on the left wrist to obtain simultaneous pulse oximetry and smartwatch pulse oximetry readings for comparison. This process was then repeated using the right index finger and wrist (Figure 1). All patients were sitting from 45-60 degrees upright, with their arm by their side on a hospital bed. Both pulse oximetry and smartwatches were applied by clinicians, ensuring a snug fit of the watch strap on the wrist of the patient. Clinically significant hypoxia was defined as an oxygen saturation reading of 92% or lower recorded on the reference device. The number of attempts to obtain a recording for each watch was collected after the enrolment of the sixty-fourth patient. If either smartwatch was unable to obtain an oximetry reading after 10 minutes, it was classified as an inconclusive measurement.

Both smartwatches were disinfected between patients using an alcohol-based disinfectant, and appropriate personal protective equipment was worn during each patient encounter. All results were electronically recorded into a secure Research Electronic Data Capture (REDCap) server, and baseline data were entered retrospectively to reduce infection transmission risk.^6^

### Statistical Analyses

Sensitivity, specificity, predictive values, and likelihood ratios were obtained using 2 × 2 contingency tables. Accuracy (A) was estimated by determining the percentage of true positive (TP) and true negative (TN) results compared with all results (R) defined as A = (TP + TN) ÷ R, with the primary analysis excluding inconclusive results. For the secondary analysis, if either smartwatch was unable to obtain an oxygen saturation reading, it was categorized as a false negative if ward-based PPG measurements reported significant hypoxia (SpO_2_ of ≤92%) and categorized as a false positive if ward-based PPG was within normal limits (SpO_2_ of >92%). The decision to treat unobtainable PPG measurements in this manner for statistical analysis was to provide a pragmatic result relevant to clinical use. Patients with hypoxia and without a PPG reading may not seek further assessment (ie, falsely reassured, therefore resulting in a false negative), whereas those with SpO_2_ > 92% without a PPG reading may still proceed to seek medical attention to confirm they are not hypoxic, and therefore result in a false positive. Continuous variables were expressed as mean ± SD and categorical data were expressed as numbers and percentages. Continuous variables were compared using ANOVA or *t* test, and categorical variables were compared using Pearson’s χ^2^ or Fisher exact tests as appropriate. Spearman rank correlation (r_s_) coefficients and Bland-Altman comparison with 95% limits of agreement (LoA) were assessed. Bias was the calculated mean difference between the smartwatch and standard PPG oximetry values. *P* values of <.05 were considered significant. Statistical analyses were carried out using Stata for Windows (Stata/MP 13.1).

## Results

During the study period, a total of 750 smartwatch pulse oximetry measurements (AW, n=379; WS, n=371) and 400 ward oximetry readings were successfully obtained from 200 patients (male 54%, age 66±18 years). The baseline and clinical characteristics are shown in Table 1. A total of 24% of patients were hypoxic at the time of assessment (n=48), and in this cohort, 77% were receiving supplemental oxygen (nasal oxygen, n=27; high flow oxygen, n=9; noninvasive ventilation, n=1). All other patients (n=163) were breathing room air when their readings were obtained.

**Table 1:** Baseline Characteristics

Characteristic	Patients With COVID-19 n=200 (%)
Mean age ± SD (y)	66±18
Male sex	108 (54%)
Mean hemoglobin (g/L)	125
Lung disease	70 (35%)
Peripheral arterial disease	39 (19.5%)
Supplemental oxygen	37 (18.5%)
Standard nasal oxygen	27 (13.5%)
High flow nasal prongs	9 (4.5%)
Noninvasive ventilation	1 (0.5%)
Fitzpatrick skin color	
Type I	32 (16%)
Type II	73 (36.5%)
Type III	66 (33%)
Type IV	17 (8.5%)
Type V	7 (3.5%)
Type VI	5 (2.5%)

For the primary analysis, when removing inconclusive readings where a smartwatch oxygen saturation reading was unable to be obtained despite multiple attempts (AW, n=21; WS, n=29), the Apple Watch reported a sensitivity and specificity of 34.8% and 97.5%, with a positive predictive value (PPV) and negative predictive value (NPV) of 78.1% and 85.6%, respectively. By contrast, the WS had a sensitivity and specificity of 68.5% and 80.8% with a PPV and NPV of 44.7% and 91.9%, respectively. The overall accuracy for the AW and WS were 84.9% and 78.5%, respectively (Table 2). Only 3 patients had inconclusive readings for both watches bilaterally. For the secondary analysis, in which inconclusive readings were included, the AW had reduced sensitivity and specificity of 32.3% and 93.2%, respectively, with a PPV of 55.2% and NPV of 84.2%. The WS also had reduced sensitivity and specificity of 62.4% and 75.4%, respectively with a PPV of 37.1% and NPV of 89.6%. Overall accuracy was 80.7% for the AW and 73% for the WS (Table 3).

**Table 2:** Detection of Clinically Significant Hypoxia With Inconclusive Measurements Excluded, Against Ward-Based PPG as Reference standard

	Sensitivity % (CI)	Specificity % (CI)	PPV %	NPV %	PLR	NLR	Accuracy %
Apple Watch Series 7	34.8 (25.2-45.4)	97.5 (95.4-98.9)	78.1	85.6	14.11	0.67	84.9
Withings ScanWatch	68.5 (58.0-77.8)	80.8 (76.6-84.5)	44.7	91.9	3.56	0.39	78.5

NLR, negative likelihood ratio; NPV, negative predictive value; PPG, photoplethysmography; PPV, positive predictive value; PLR, positive likelihood ratio.

**Table 3:** Detection of Clinically Significant Hypoxia, Including Inconclusive Measurements, Against Ward-Based PPG as Reference Standard

	Sensitivity % (CI)	Specificity % (CI)	PPV %	NPV %	PLR	NLR	Accuracy %
Apple Watch Series 7	32.3 (23.3-42.5)	93.2 (90.2-95.5)	55.2	84.2	4.75	0.73	80.7
Withings ScanWatch	62.4 (52.2-71.8)	75.4 (71.1-79.4)	37.1	89.6	2.54	0.50	73.0

NLR, negative likelihood ratio; NPV, negative predictive value; PPG, photoplethysmography; PPV, positive predictive value; PLR, positive likelihood ratio.

**Figure 2:**
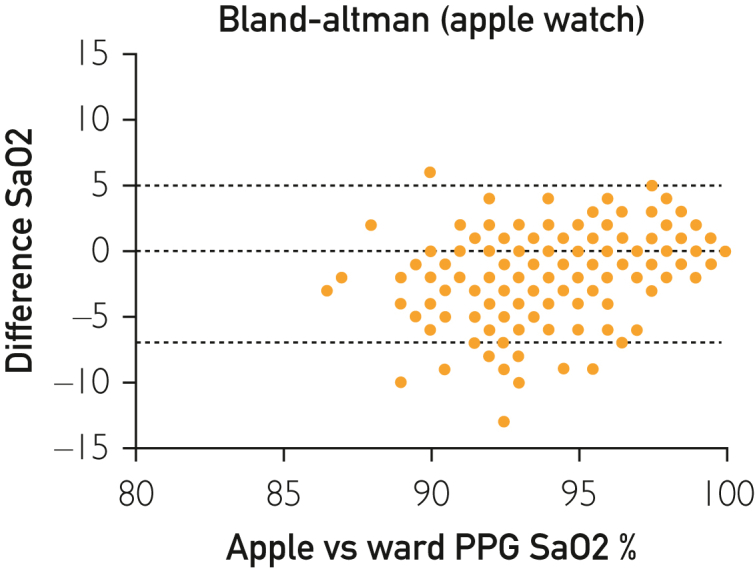
Bland-Altman analysis for the Apple Watch.

**Figure 3:**
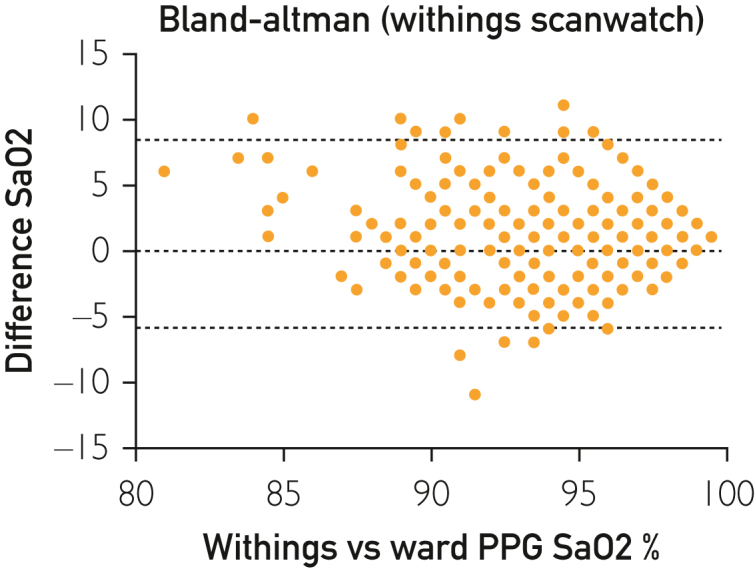
Bland-Altman analysis for the Withings ScanWatch.

Bland-Altman analysis for the AW reported a −0.78 bias with a wide 95% LoA (−6.0% to 4.5%) (Figure 2). The Withings cohort revealed a 1.23 bias with a wide 95% LoA (−5.3% to 7.9%) (Figure 3). When assessing only patients with significant hypoxia, the AW had a bias −4.3 (95% LoA: −10.2 to 1.6) compared with the WS bias of −1.2 (95% LoA: −9.0 to 6.7). For patients within normal oxygen saturation range, the AW had a bias −0.2 (95% LoA: −4.3 to 3.9) compared with the WS bias of −1.7 (95% LoA: −4.5 to 8.0).

Overall, both watches found only moderate correlation to pulse oximetry readings analyzed using Spearman rank correlation coefficients, with the AW exhibiting increased correlation (AW: r_s_=0.61; WS: r_s_=0.51, both *P*<.001).

From an analysis of 136 patients, we revealed an average (SD) of 3.7 (±2.2) and 4.4 (±2.7) attempts respectively to successfully obtain bilateral measurements from the AW and WS (in which 1 attempt indicates successful PPG readings bilaterally on the first measurement).

## Discussion

To our knowledge, this is the first prospective study which evaluates the accuracy of inbuilt PPG in the AW Series 7 and WS in patients with COVID-19. Our results report that when compared with reference standard PPG technology, both smartwatches have reduced sensitivity in detecting clinically significant hypoxia in patients hospitalized with COVID-19 infection. Although an improvement in sensitivity, specificity, PPV, NPV and accuracy were seen when inconclusive readings were excluded, a real-world outpatient setting may result in many consumers avoiding further attempts to obtain a smartwatch oximetry trace and instead seek medical review, increasing the clinical load and burden on hospital departments. However, our study was performed in an inpatient cohort, and these results may not be applicable to those in a community or ambulatory care setting.

Current guidelines suggest monitoring of oxygen saturations for those managing COVID-19 infection at home however they do not specify the types of devices that can be used to provide these measurements.^3^ Due to the ease of accessibility and the ubiquity of smartwatches in a global population, many consumers will elect to use their device to monitor oxygen saturations instead of attending a medical facility or purchasing a dedicated oximeter. However, there are few published studies that have assessed the accuracy of these watches in patients with relevant acute respiratory disease. Although some studies have assessed smartwatch oximetry accuracy in patients with chronic obstructive pulmonary disease, interstitial lung disease and cardiovascular disease, these are not large prospective trials and did not examine patients also infected with COVID-19.^7^, ^8^, ^9^ An early study performed by Pipek et al^7^ revealed that in patients with interstitial lung or chronic obstructive pulmonary disease, the AW found a strong positive correlation with both SpO_2_ and heart rate measurements. Although we did not assess the accuracy of smartwatch heart rate measurements in our patient cohort, our results identify a discrepancy in smartwatch oximetry readings and ward-based PPG, which has not been described in the previous literature. Similar results were also identified by Spaccarotella et al^8^, with good concordance found with smartwatch SpO_2_ measurements when compared with standard commercial devices. Although this study was similar to the current study in terms of ethnicity and Fitzpatrick skin color, it included healthy controls, patients with pulmonary or cardiovascular disease rather than patients with COVID-19 in the current study.^8^ Finally, studies that have reported a positive correlation in smartwatch oximetry measurements in patients with hypoxia revealed high rates of inconclusive readings. A study performed by Kirszenblat et al^9^ assessed the Withings smartwatch in patients with varying levels of hypoxia, and while they did report adequate accuracy compared with arterial blood gas oximetry readings, 15%-24% of WS oximetry results were inconclusive, which parallels our findings. The underlying cause for the discrepancy in measurements is difficult to ascertain but could be attributed to the different underlying method of SpO_2_ acquisition between these devices. Most commercial and hospital-grade peripheral oximetry monitors obtain SpO_2_ tracings using transmissive pulse oximetry, in which different wavelengths of light are passed through a measurement site to a photodetector, which identifies a change in wavelengths due to the absorption of light.^10^ In comparison, smartwatch devices obtain SpO_2_ measurements by reflective pulse oximetry, which uses a light detector generally located adjacent to the emitter to measure backscatter of light.^11^ Although both methods of SpO_2_ acquisition have been studied reporting minimal differences in measurements, this difference may cause reading discrepancies in specific disease states and has not been evaluated in patients with active COVID-19 infection.

In the context of the ongoing COVID-19 pandemic and relevant strain on acute health care access, many clinicians are electing to manage patients infected with mild-to-moderate COVID-19 at home with telehealth. However, monitoring for deterioration can be difficult, with a considerable proportion of patients developing severe resting hypoxia with minimal symptoms before clinical deterioration.^12^ On the basis of our findings, both smartwatches were unable to provide sufficient accuracy for clinical use of hypoxia detection, but it is unknown whether these findings would translate to an outpatient setting with less unwell patients. For patients with COVID-19 with minimal symptoms, the NPVs obtained for both devices may provide some level of clinical reassurance that home management remains appropriate. However, in patients with relevant respiratory symptoms but a borderline or normal watch oxygen saturation reading, the low sensitivity (32.3% and 64.2% for AW and WS, respectively) means that some patients may be falsely reassured, potentially delaying early medical intervention. Although this risk of delayed assessment and intervention is generally present in patients with COVID-19 relying on symptom identification to recognize the need for medical review, some consumers may use their smartwatch as an additional form of reassurance. Conversely, if translated into clinical use, the low PPV would lead to a considerable number of unnecessary presentations for clinical assessment when the patient is not hypoxic, putting unnecessary strain on the health care system. From our results, the high false positive rate would lead to more than half of all patients recording hypoxia, prompting further medical attention.

Our study also highlights the multitude of factors associated with obtaining an accurate watch PPG tracing. All measurements were performed on inpatients with COVID-19 infection with guidance and supervision from medical staff. Despite this, 3 patients had inconclusive readings from both watches bilaterally, with these patients having a history of lung disease, peripheral arterial disease, or morbid obesity, respectively. Most patients that had inconclusive readings generally had at least 1 characteristic associated with difficult acquisition of measurements (lung disease, peripheral arterial disease, Fitzpatrick skin type > 3, and morbid obesity). Of the remaining patients without these characteristics, 2 patients also had COVID-19 infection complicated by pulmonary embolism, and 2 patients had concomitant alcohol withdrawal. It is unclear whether the latter group had inconclusive readings secondary to a tremor, associated liver pathology, such as hepatopulmonary syndrome, or undiagnosed lung or peripheral arterial disease. It was noted early in the study that even with direct assistance, it took ∼4 attempts on average to obtain a smartwatch reading. The slightly higher average attempt rate to obtain PPG readings from the WS could be attributed to the increased time to acquisition (30 seconds) compared with the AW (15 seconds), as minimal movement of the patient’s hand or arm resulted in restarting the measurement recording. In addition, hospitalized patients with COVID-19 may be more unwell or frail compared with the general population, resulting in more movement and measurement artefact, and subsequently impair the ability of both smartwatches to obtain oximetry readings. Previous studies have also used an average of 3 readings per patient to improve device accuracy, however as our study was analyzing 2 watches, in addition to unsuccessful attempts this would increase total attempts to approximately 10 per patient. Therefore, for patient comfort and to reduce unnecessarily prolonged exposure of the clinicians to the COVID-19 positive patients, we elected to obtain 2 readings for each watch per patient.

### Limitations

Our study has some limitations. Only patients with active COVID-19 infection requiring hospital admission were recruited; these results may not be extrapolatable to patients with mild COVID-19 infection who do not require hospital admission, or to non-COVID–19 positive settings, such as future novel respiratory virus outbreaks. Hospitalized patients are also more likely to have multiple comorbidities. Patients were directly supervised and instructed when obtaining smartwatch PPG tracings, and the accuracy of our findings may differ from a nonsupervised community setting. Although initial smartwatch heart rate monitoring could be used to help ensure patient compatibility with the device to obtain oximetry readings, our study is attempting to analyze pragmatic, real-world scenarios, in which patients and consumers should expect their smartwatch to provide an immediate oximetry reading, and not have to troubleshoot their device. Although used as the reference standard in this study, the accuracy of hospital PPG monitors in patients with active COVID-19 infection has recently been questioned, which could affect the accuracy of the comparative analyses performed.^13^ In healthy patients, both hospital and smartwatch oximetry have an acceptable SpO_2_ variance of 2%-3% and this difference could affect the classification of measurements into hypoxic and normoxic categories and subsequently may affect our statistical findings in Table 2 and 3. However, to address this issue, we have incorporated Bland-Altman analyses to report bias and provide upper and lower LoA, similar to validation studies performed by both Apple Inc and Withings.^14^ Finally, nearly all patients enrolled in this study had relatively fair skin (Fitzpatrick skin types I, II, and III). Our study results may not be generalizable to all Fitzpatrick skin types and our sample size was insufficient to perform a comparative analysis.

## Conclusion

Smartwatches are able to provide SpO_2_ readings; however, compared with the reference standard ward-based pulse oximetry, their overall accuracy may not be sufficient to replace standard PPG technology in monitoring hypoxia in patients with COVID-19 who are highly symptomatic or have underlying conditions predisposing them to a higher risk of clinical deterioration.

## Potential Competing Interests

The authors report no conflicts of interest.
